# Case Report: Two episodes of hyperinflammation in an infant consistent with multisystem inflammatory syndrome in children: recurrence or rather two different diseases?

**DOI:** 10.3389/fped.2025.1559244

**Published:** 2025-06-05

**Authors:** Markus-Johann Dechant, Holger Michel, Michael Kabesch, Michael Melter, Stephan Gerling

**Affiliations:** University Children’s Hospital Regensburg (KUNO-Clinics), University of Regensburg, Regensburg, Germany

**Keywords:** hyperinflammation, infant, recurrence, multisystem, inflammatory, case report

## Abstract

Multisystem inflammatory syndrome in children (MIS-C) is a hyperinflammatory disease that occurs after infection with severe acute respiratory syndrome coronavirus 2 (SARS-CoV-2). We report a 9-month-old male infant with two episodes of hyperinflammatory disease, each involving the coronary arteries, within a short period of time during the SARS-CoV-2 pandemic. Although both episodes met the official criteria for MIS-C, this case illustrates the difficulty in distinguishing MIS-C from its main differential diagnosis, Kawasaki disease (KD). Recurrence of postviral hyperinflammatory disease is rare. Compared with KD, the recurrence of MIS-C is even rarer, but clinicians should be aware of this possibility. Our case also emphasizes the need to follow up these patients closely and to detect sequelae regularly, especially cardiovascular sequelae, at an early stage.

## Introduction

A new rare hyperinflammatory disease, now known as multisystem inflammatory syndrome in children (MIS-C), emerged shortly after the beginning of the severe acute respiratory syndrome coronavirus 2 (SARS-CoV-2) pandemic (1). Its main characteristics are fever, elevated inflammatory parameters, and multiple organ dysfunction, in particular cardiac dysfunction and severe shock, and hyperinflammation is considered the presumed cause. MIS-C shares similarities with Kawasaki disease (KD), but epidemiological, clinical, and immunological differences may help to distinguish MIS-C from KD (2, 3). The clinical course of children affected by MIS-C tends to be more severe and may be potentially life-threatening, requiring a rapid diagnosis and prompt treatment (4). Complete recovery without sequelae is the usual course even with severe initial presentations (5). We describe a male infant who had two episodes of hyperinflammatory disease, each involving the coronary arteries, within a short period of time during the SARS-CoV-2 pandemic and discuss the difficulty in distinguishing MIS-C from KD.

## Case report

### First episode

**Figure 1:**
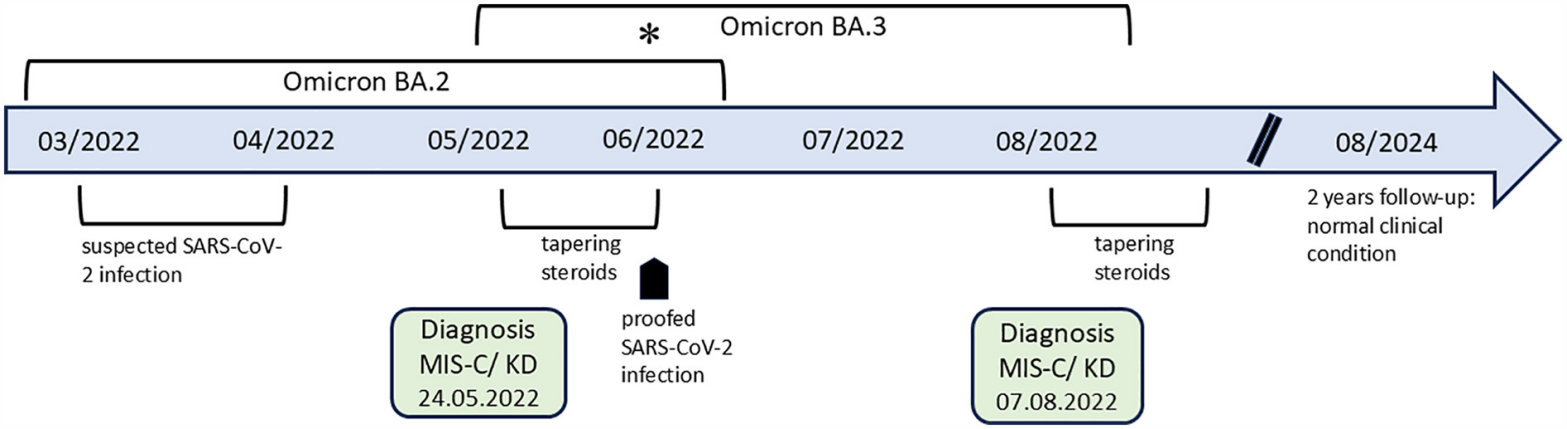
The timeline shows the course of both episodes. There was a change of SARS-CoV-2 Omicron subtype in Bavaria, with a predominance of variant BA.3 (supposed second infection) compared to variant BA.2 (supposed first infection), indicated with an asterisk.

A previously healthy 9-month-old male infant, up to date with routine immunizations (without the SARS-CoV-2 vaccine, as it was not yet licensed for this age group), presented to our emergency department with a fever of up to 39.8°C for 8 days, accompanied by rhinorrhea. Prior to this presentation, oral antibiotic therapy with amoxicillin/clavulanic acid had been initiated by the general pediatrician, which was given for 3 days without clinical improvement. Diarrhea and vomiting had also been present for 3 days. On presentation, the infant appeared ill with low-grade fever, tachycardia, and normal room air oxygen saturation, otherwise, there were no further indicators on physical examination to explain the persistent fever. The absence of symptoms consistent with KD was notable, including, but not limited to, extremity changes, skin rash, conjunctivitis, oral changes, or cervical lymphadenopathy. Further diagnostics based on the current medical history and current clinical symptoms are shown in Table 1, which were unremarkable. Due to the persistent fever, non-response to antibiotics, and gastrointestinal symptoms, the presence of a hyperinflammation syndrome was suspected. The SARS-CoV-2 anti-spike antibodies were slightly positive (12.5 U/ml, normal range 0–0.79 U/ml), whereas the anti-nucleocapsid antibodies were negative, yet no prior history of infection or exposure to an infected individual was documented. His echocardiogram showed normal biventricular function but small aneurysms of the left main coronary artery (2.6 mm, *Z*-score +2.6) and left anterior descending coronary artery (2.2 mm, *Z*-score +2.8) (6). The following day, fever persisted (day 9) and there was a further increase in D-Dimers and N-terminal pro B-type natriuretic peptide (NT-proBNP). Based on these findings, the abnormal laboratory findings (Table 2), and the revised MIS-C definition by the Centers for Disease Control and Prevention (CDC) (7), MIS-C was considered the most likely differential diagnosis in the context of the global SARS-CoV-2 pandemic. In light of this, according to the Bavarian State Office for Health and Food Safety (LGL), 109,284 confirmed new SARS-CoV-2 infections were reported in Bavaria in the presumed week of infection (week 16 of the calendar year). The predominant type was Omicron BA.2 and the highest number of confirmed new infections was in week 12, with 309,345 infections (8). However, based on the findings, there was a possibility that incomplete KD (IKD) may also be a differential diagnosis (Table 3). According to the current guidelines, immunomodulatory therapy with intravenous immunoglobulins (2 g/kg) and prednisolone (2 mg/kg divided into three single doses) and anti-inflammatory therapy with acetylsalicylic acid (40 mg/kg divided into four single doses) were started (9). There was a good response to therapy with rapid normalization of body temperature, improvement of general condition, inflammatory markers, and resolved coronary artery aneurysms. The patient was discharged on the fifth day after admission with acetylsalicylic acid (5 mg/kg once a day) for antiplatelet effect. At the regular follow-up visits after 3 and 6 weeks, he presented in a good clinical condition with normal cardiac findings. No signs of skin peeling were observed. The steroid therapy was tapered for 20 days and the acetylsalicylic acid was stopped after 6 weeks. Importantly, between the first and second follow-up visits, the patient had a polymerase chain reaction (PCR)-proven SARS-CoV-2 infection with mild symptoms of an upper respiratory tract infection (Figure 1).

**Table 1:** Additional diagnostic laboratory studies for each episode.

Study	First episode May 2022	Second episode August 2022
Infectious diseases		
Nasopharyngeal study		
Influenza A/B	Negative	Negative
RSV	Negative	Negative
Stool study		
Rotavirus	Negative	Negative
Norovirus	Negative	Negative
Adenovirus	Negative	Negative
Enterovirus		Negative
Bacteria		Normal flora
Serum Study		
EBV nuclear antibody IgG		Positive
EBV anti-virus capsid antigen IgM		Negative
EBV anti-virus capsid antigen IgG		Positive
Urine analysis	Negative	Negative
Blood culture	Negative	Negative
Imaging		
Chest x-ray	Negative	Negative
Abdominal sonography		Unremarkable

EBV, Epstein–Barr virus; RSV, respiratory syncytial virus, IgG, Immunoglubulin G; IgM, immunoglobulin M.

**Table 2:** Significant laboratory values during both episodes of hyperinflammation.

Laboratory study (reference range)	First episode May 2022	Second episode August 2022
White blood cells (6,000–17,500 cells/mm^3^)	22,500	20,700
Hemoglobin (10.8–12.8 g/dl)	9	9.3
Platelets (150,000–450,000 cells/mm^3^)	984,000	719,000
Absolute neutrophil count (1,500–8,700 cells/mm^3^)	13,000	7,600
Absolute lymphocyte count (3,000–10,000 cells/mm^3^)	7,700	7,400
Sodium (129–145 mmol/L)	129	137
Albumin (3.7–5.3 g/dl)	2.92	3.61
Aspartate aminotransferase (10–82 U/L)	26	37
Alanine aminotransferase (10–33 U/L)	21	31
C-reactive-protein (1–5 mg/L)	44	157
Ferritin (20–200 ng/ml)	77	161
D-Dimer (0–500 ng/ml)	1,496	945
Fibrinogen (150–400 mg/dl)	513	
Troponin t-hs (0–14 ng/L)	5	<3
NT-proBNP (<100 pg/ml)	1,222	1,668
SARS-CoV-2 PCR	Negative	Negative
Anti SARS-CoV-2 Spike (0–0.79 U/ml)	12.5	
Anti SARS-CoV-2 Nucleocapsid (negative)	Negative	
Time interval between onset of fever and lab results (days)	9	4

hs, highly sensitive; NT-proBNP, N-terminal pro B-type natriuretic peptide; SARS-CoV-2, severe acute respiratory syndrome coronavirus 2; PCR, polymerase chain reaction.

**Table 3:** Comparison of the adapted multisystem inflammatory syndrome in children case definition (7) and adapted incomplete Kawasaki disease case definition (17) and the findings in the patient.

MIS-C^a^^,^^b^	First episode May 2022	Second episode August 2022
Clinical criteria (all of them)		
Subjective or documented fever	Positive	Positive
Clinical severity requiring hospitalization	Positive	Positive
Evidence of systemic inflammation	Positive	Positive
At least two new organ manifestations		
Cardiac involvement	Positive	Positive
Gastrointestinal involvement	Positive	Positive
Laboratory criteria		
Detection of SARS-CoV-2 RNA in a clinical specimen up to 60 days before or during hospitalization	Negative	Positive
Detection of SARS-CoV-2-specific antibodies in serum, plasma, or whole blood associated with current illness resulting in or during hospitalization	Positive	Not tested
Epidemiologic linkage criteria		
Close contact with a confirmed or probable case of COVID-19 disease in the 60 days before hospitalization	Negative	Positive
IKD^b^		
Child with fever for ≥5 days and two or three compatible clinical criteria	Negative	Negative
Infant with fever for ≥7 days without other explanation	Positive	Negative
Evidence of systemic inflammation	Positive	Positive
Three or more of the laboratory findings	Positive	Positive
Positive echocardiogram	Positive	Positive

MIS-C, multisystem inflammatory syndrome in children; IKD, incomplete Kawasaki disease; WBC, white blood count; COVID-19, coronavirus disease 2019.

^a^ Confirmed: meets the clinical and the confirmatory laboratory evidence.

^b^ Only the positive findings of each case definition were mentioned.

### Second episode

The patient presented again to our Emergency Department 3 months after being discharged from the hospital with fever and decreased general condition. The fever had persisted for 4 days and was resistant to oral antibiotic therapy with amoxicillin/clavulanic acid administered for 2 days. As already mentioned, the patient had a PCR-proven SARS-CoV-2 infection with mild symptoms 7 weeks before. On presentation, the infant was ill with fever, tachycardia, and diarrhea. The clinical examination revealed a reddened ear on the right side and a reddened throat, otherwise, no other indicators could be found. Comparable to the first episode, the absence of symptoms consistent with KD was again notable. Further diagnostics based on the current medical history and current clinical symptoms are shown in Table 1, which were unremarkable, with the exception of a positive Epstein–Barr virus (EBV) serology. The initial echocardiography was normal and the antibiotic therapy was continued. The patient showed no clinical improvement, but inflammatory markers and NT-proBNP increased (Table 2). Serial echocardiography again revealed a renewed small aneurysm of the left main coronary artery (2.7 mm, *Z*-score +2.6) (6). Thus, a second episode of hyperinflammatory disease was again considered the most likely diagnosis (Table 3). At the time of confirmed infection, 7 weeks before the second episode, in week 25 of the calendar year, 79,051 new infections were reported in Bavaria. In comparison to the first episode, the predominant variant had changed to Omicron BA.3 and the highest number of new infections was in week 29, with 130,676 infections (8). An immunomodulatory therapy and platelet aggregation inhibition with the same regimen as in the first episode was started and he showed a prompt clinical improvement to therapy. No signs of skin peeling were observed. Until now, follow-up care has included a period of more than 2 years after diagnosis of the second episode with normal clinical and echocardiographic findings (most recently left main coronary artery 2.7 mm, *Z*-score +1.6 and left anterior descending coronary artery 1.8 mm, *Z*-score +0.51) (Figure 1) (6).

## Discussion

We report a case of recurrent hyperinflammation in an infant and highlight the challenge of distinguishing between MIS-C and KD/IKD. Recurrences of KD are rare. A comparison of data from the United States and Japan showed a recurrence rate of 1.7% in the United States, rising to 3.5% in Asians and Pacific Islanders (10). Thus far, there have been few reports on the worsening of symptoms in patients with suspected MIS-C after initial clinical improvement due to immunomodulatory therapy and both cases can be characterized as rebounds (11, 12). Another study described patients who had undergone MIS-C who did not develop MIS-C again after SARS-CoV-2 reinfection (13). Importantly, one case report presented a 9-year-old boy with two distinct illnesses that occurred within a few months, which were both rated as MIS-C, and therefore, a recurrence of the disease was considered (14). This case highlights, on the one hand, the different possible phenotypes of MIS-C and, on the other hand, the possibility that a subset of children may have a predisposition for repeat MIS-C. Another reason for recurrence, or perhaps in parallel, could be the different virulence of the SARS-CoV-2 subtypes triggering MIS-C.

It has been proposed by several studies that specific risk factors may play a role in the development of MIS-C. These include epidemiological factors, such as age and gender; pre-existing conditions, such as asthma and obesity; and congenital immune deficiencies (15, 16).

Meeting the criteria of the MIS-C definition by the CDC may be challenging, especially if not all typical clinical signs of a disease are present or when the clinical pictures are similar, as in KD/IKD and MIS-C. This represents a particular challenge during infancy when persistent fever is the sole clinical finding or when there are subtle or fleeting clinical signs in addition to fever. Therefore, initiating adequate therapy may be delayed (17). These circumstances put the patient at risk of disease progression or sequelae. The CDC requires detection or contact with SARS-CoV-2 as one crucial point in their diagnostic criteria for MIS-C (7). In the initial episode, antibodies against the spike protein were detected at a low level, while those against the nucleocapsid were negative. It is important to consider the following when evaluating this finding. The patient was not breastfed and the mother had neither a proven SARS-CoV-2 infection nor a SARS-CoV-2 vaccination before or during pregnancy. Therefore, it appears conceivable that the positive serology against a previous SARS-CoV-2 infection was not transmitted transplacentally, with uncertainty regarding a possible maternal sub-clinical SARS-CoV-2 infection. Furthermore, we interpreted the positive EBV serology found during the second episode as transferred antibodies due to the immunoglobulins that had been administered 3 months earlier. It is possible that maternal antibodies could be a factor, however, it should be noted that the mother did not appear to have experienced an EBV infection, at least from a clinical perspective. Although we have no proof of this, it would be unusual for maternal antibodies to persist until a child reaches the age of 12 months. One would actually expect that the antibodies would be higher as MIS-C develops within 2–6 weeks after contact or active infection with SARS-CoV-2. However, in a matched analysis of convalescent subjects, anti-nucleocapsid IgG levels fell significantly over time, with 8% of subjects being seronegative at 5 weeks, 34% at 6 months, and 48% at 12 months (18). In addition, in a study conducted in regions of Germany with a relatively high incidence of SARS-CoV-2 infections, a few patients who tested positive by PCR showed no seroconversion (19). Therefore, the absence of antibodies against the nucleocapsid antigen and even the low level of antibodies against the spike protein may not exclude a recent infection with SARS-CoV-2.

Symptoms of hyperinflammation reappeared in the patient more than 6 weeks (44 days) after the end of steroid therapy for the first episode. In between, he was in a normal clinical condition, including normal echocardiographic findings during two follow-up visits, but experienced a symptomatic infection with SARS-CoV-2, proven by PCR, 7 weeks before the second episode. The CDC definition and criteria led us to suspect a possible second episode of MIS-C, but, similar to the first episode, incomplete Kawasaki disease may also be a possibility (Table 3). SARS-CoV-2 antibodies were not determined because of the previously proven SARS-CoV-2 infection. In addition, it should be noted that the detection of SARS-CoV-2 antibodies would generally become less important as population immunity increases due to ongoing exposure or vaccination. This makes seropositivity less specific as a diagnostic marker for MIS-C (20). The reason for the possible SARS-CoV-2 reinfection within a few months may be due to the following. In the time period between the possible first asymptomatic and the proven second symptomatic infections, there was a change in the Omicron subtype as mentioned above (Figure 1). The Omicron variants contained many new mutations in the spike protein, which caused a rapid spread around the world and large outbreaks among children and adolescents (21). Furthermore, the second infection occurred during the period when the patient was still receiving systemic steroid therapy in a tapering form. Steroids may influence the dysregulated immune response in a positive way but may exert inhibitory effects on the immune defense (22). In addition, it needs to be considered that infants generally have a different and less effective humoral immune response than older children following natural infections. This may result from the inability of bone marrow at this age to establish a pool of long-lived plasma cells (23). All these reasons make the body more susceptible and can contribute to possible reinfection.

We admit that, in both episodes, IKD has to be considered as the most likely differential diagnosis (Table 3). This is supported by the values of some laboratory parameters, such as lymphocytes and thrombocytes, which are considered important differentiators between MIS-C and KD/IKD, and the age of our patient, which is in contrast to most reported patients (1, 2). A reliable distinction between MIS-C and KD/IKD would be of great importance as the diagnoses have different risks of cardiovascular involvement and clinical decompensation. In light of this, there is ongoing focus on leveraging various tools, such as biomarkers, to enhance this differentiation. For example, an analysis was conducted by the working group of the International Kawasaki Disease Registry (IKDR) of a substantial and varied cohort of patients suffering from KD and MIS-C, with the objective of identifying disparities in cardiac biomarkers. It was demonstrated that higher NT-proBNP (≥1,500 ng/L) and troponin I (≥20 ng/L) values are indicative of MIS-C compared to KD with reasonable specificity and may be clinically useful in differentiation (24). In our case, this would be applicable in the context of the second episode. Echocardiography represents a further point at which these two diseases can differ with regard to the cardiovascular system. Coronary artery involvement is more prevalent in patients with KD/IKD, while heart valve involvement, left ventricular systolic dysfunction, and pericardial effusion are more prevalent in MIS-C (25). Another possible way to differentiate between MIS-C and KD was demonstrated using circulating endothelial cells, a marker for inflammatory vessel wall damage. The amount of circulating endothelial cells was significantly higher in KD compared to MIS-C. One possible explanation for this could be that the changes in the vessel walls in MIS-C are secondary to systemic inflammation, possibly due to a cytokine storm, and not necrotizing arteritis as observed in KD (26). In addition, it has been shown that the determination of immune profiles can facilitate the differentiation between these two diseases, but, similar to the determination of circulating endothelial cells, this was not possible in our institution at the time of presentation (27). However, no specific laboratory marker exists for either MIS-C or KD and MIS-C has been identified in infants who appear to have a less critical clinical course than older children (28, 29). Therefore, we argue that after a thorough workup for alternative diagnoses (Table 1); a failure to improve on antibiotics; coronary artery aneurysms, which are very rarely described in other circumstances; and rapid improvement after immunomodulatory treatment, a diagnosis of recurrence of hyperinflammation can be made in the absence of clear evidence of MIS-C or IKD (30, Table 3).

## Conclusion

This case report aims to raise awareness of the potential for recurrence of hyperinflammation. It also highlights the fact that the current diagnostic criteria for MIS-C may not accurately identify all affected children adequately and the primary challenge in this clinical case was to make an accurate diagnosis. This is especially the case in infants, in whom the clinical course and the course of laboratory parameters may differ from older children. Further, it should be highlighted that despite two episodes of a hyperinflammatory disease within a short period of time, there was a good response to an immunomodulatory therapy with rapid clinical improvement and normalization of the affected coronary arteries. There is still uncertainty about the long-term course of hyperinflammation that occurred during the SARS-CoV-2 pandemic. Therefore, to enhance our knowledge in depth, it is necessary that these patients are followed up closely and regularly to detect sequelae, especially cardiovascular sequelae, at an early stage.

## Data Availability

The original contributions presented in the study are included in the article/Supplementary Material, further inquiries can be directed to the corresponding author.
